# Artificial Intelligence in the Diagnosis and Quantitative Phenotyping of Hyperkinetic Movement Disorders: A Systematic Review

**DOI:** 10.3390/jcm13237009

**Published:** 2024-11-21

**Authors:** Joaquin A. Vizcarra, Sushuma Yarlagadda, Kevin Xie, Colin A. Ellis, Meredith Spindler, Lauren H. Hammer

**Affiliations:** 1 Department of Neurology, Perelman School of Medicine, University of Pennsylvania, Philadelphia, PA 19104, USA; 2 Parkinson’s Disease Research, Education and Clinical Center, Philadelphia Veterans Affairs Medical Center, Philadelphia, PA 19104, USA; 3 Center for Neuroengineering and Therapeutics, University of Pennsylvania, Philadelphia, PA 19104, USA; 4 Department of Neurology, Emory University School of Medicine, Atlanta, GA 30322, USA; 5 Department of Bioengineering, School of Engineering and Applied Sciences, University of Pennsylvania, Philadelphia, PA 19104, USA

**Keywords:** machine learning, systematic review, artificial intelligence, movement disorders, deep learning, tremor, chorea, ataxia, tics, dystonia

## Abstract

**Background**: Hyperkinetic movement disorders involve excessive, involuntary movements such as ataxia, chorea, dystonia, myoclonus, tics, and tremor. Recent advances in artificial intelligence (AI) allow investigators to integrate multimodal instrumented movement measurements and imaging techniques and to analyze these data together at scale. In this systematic review, we aim to characterize AI’s performance in diagnosing and quantitatively phenotyping these disorders. **Methods**: We searched PubMed and Embase using a semi-automated article-screening pipeline. **Results**: Fifty-five studies met the inclusion criteria (n = 11,946 subjects). Thirty-five studies used machine learning, sixteen used deep learning, and four used both. Thirty-eight studies reported disease diagnosis, twenty-three reported quantitative phenotyping, and six reported both. Diagnostic accuracy was reported in 36 of 38 and correlation coefficients in 10 of 23 studies. Kinematics (e.g., accelerometers and inertial measurement units) were the most used dataset. Diagnostic accuracy was reported in 36 studies and ranged from 56 to 100% compared to clinical diagnoses to differentiate them from healthy controls. The correlation coefficient was reported in 10 studies and ranged from 0.54 to 0.99 compared to clinical ratings for quantitative phenotyping. Five studies had an overall judgment of “low risk of bias” and three had external validation. **Conclusion**: There is a need to adopt AI-based research guidelines to minimize reporting heterogeneity and bolster clinical interpretability.

## 1. Introduction

Hyperkinetic movement disorders are neurological conditions characterized by excessive, involuntary movements, and include ataxia, chorea, dystonia, myoclonus, tics, and tremor. Historically, diagnosis of such disorders has relied on the appreciation of an abnormal movement’s *phenomenology*, or the science and art of classifying abnormal movements based on the clinician’s physical exam [1]. Diagnosis therefore relies on subjective assessment and may vary based on the clinical neurology training of the clinician (e.g., diagnostic assessment may differ between a primary care physician and a movement disorders specialist). The question remains whether objective, quantitative assessments could aid in diagnosing and phenotyping these conditions. In recent years, research has attempted to instrument movement measurement with body-worn sensors (e.g., accelerometers, gyroscopes), video and audio analysis, or electrophysiology (e.g., electromyography, EMG; electroencephalography, EEG) [2]. Other data modalities, such as neuroimaging (e.g., anatomical or functional MRI), can also be interrogated to identify specific disease signatures [3]. These methods lead to large amounts of data that are challenging to interpret with traditional computational methods [2].

Artificial intelligence (AI)-based methods offer the ability to process large datasets with variable data types (e.g., clinical assessments, genetics, imaging, electrophysiology), making them increasingly utilized in research focusing on the diagnosis and quantitative phenotyping of hyperkinetic movement disorders. AI is the broader field of computer science focused on creating systems that can perform tasks requiring human-like intelligence, such as reasoning and problem-solving. Within AI, Machine Learning (ML) enables computers to learn from data and improve over time without explicit programming. A subset of ML, Deep Learning (DL) utilizes deep neural networks with multiple layers to model complex patterns, making it particularly effective for tasks like image and speech recognition [4]. Despite the growing number of research articles describing the promises of AI tools for hyperkinetic movement disorders, clinical implementation lags behind. Several barriers to clinical translation exist, but at the most elemental level, the major barriers are the quality, interpretability, and clinical applicability of research results.

This review aims to 1. characterize the existing AI-based research in hyperkinetic movement disorders for disease diagnosis and quantitative phenotyping and 2. evaluate its quality.

## 2. Methods

This systematic review was conducted per the Preferred Reporting Items for Systematic Reviews and Meta-analyses (PRISMA) guidelines [5]. This study was conducted in accordance with the principles outlined in the Declaration of Helsinki, as revised in 2013.

### 2.1. Search Methods

We searched PubMed and Embase for peer-reviewed studies published up to December 2023 using a combination of free text and MeSH (Medical Subject Headings) for the following AI keywords: Artificial Intelligence, Machine Learning, Deep Learning, Supervised Learning, Unsupervised Learning, Reinforcement Learning, Neural Network, Natural Language Processing, Computer Vision, Data Mining, Predictive Analytics, Big Data, Feature Engineering, Model Training Algorithm; and movement disorders keywords: Tremor, Movement Disorder, Parkinson, Dystonia, Huntington, Essential Tremor, Ataxia, Myoclonus, Progressive Supranuclear Palsy, PSP, MSA, Tics (Supplementary Table S1). No restrictions were applied to gender, age, ethnicity, disease duration, or disease severity. Hypokinetic movement disorders, namely Parkinson’s disease (PD) and related conditions, were included in the search terms as they can manifest with tremor.

### 2.2. Inclusion and Exclusion Criteria

Articles were included if they utilized AI algorithms in hyperkinetic movement disorders, namely ataxia, chorea, dystonia, tics, and tremor, specifically involving disease detection and or quantitative phenotyping. Studies of PD participants were excluded if PD was the primary group of interest, as to only include studies focusing on hyperkinetic movement disorders that used PD as a comparison cohort. Articles in non-human subjects or without an English-language translation were excluded. Reviews and conference papers were excluded.

### 2.3. Selection of Studies

An article screening code was created in Python and finetuned with assistance from a free, online generative language processing model (ChatGPT-4). The program executed the following functions: a. title labeling for AI and movement disorders keywords; b. duplicate removal; c. inclusion suggestion for studies that included a combination of at least one of the AI keywords and one of the movement disorders keywords in their title. As an example, a hypothetical study titled “Diagnostic Markers of Essential Tremor identified with Machine Learning Algorithms”, would have the movement disorder keyword “Essential tremor” and the AI keyword “Machine learning” identified, and since both were present, it would be selected for inclusion. Two authors (JAV, SY) manually reviewed abstracts of the studies selected for inclusion. Twenty percent of the studies recommended for exclusion were reviewed randomly by one author (JAV) to ensure the accuracy of the title screening process. No excluded articles were reclassified as included after the manual revision. The code is freely available on the author’s GitHub (https://github.com/VizcarraJA/sysrevs_screener). The reference lists of selected articles were additionally screened for pertinent studies not included in the original search strategy.

### 2.4. PICO Questions and Data Analysis

The following PICO (Population, Intervention, Comparison, Outcome) questions were studied:

1. In studies of hyperkinetic movement disorders, what is the performance of artificial intelligence models compared to traditional non-artificial intelligence methods for disease diagnosis?
2. In studies of hyperkinetic movement disorders, what is the performance of artificial intelligence models compared to traditional non-artificial intelligence methods for quantitative phenotyping?

### 2.5. Data Extraction, Quality Assessment, and Risk of Bias

The following data were extracted from eligible studies using a standardized form: title, authors, publication year, DOI, review question, population studied, number of cases, number of controls, AI method, reference test, outcomes, type of internal validation, and type of external validation. For PICO question #1, disease diagnosis (i.e., classification task), we extracted accuracy; if accuracy was unavailable, we extracted the area under the receiver operator characteristic curve (AUROC). For PICO question #2, quantitative phenotyping, we extracted the coefficient of correlation (r) or coefficient of determination (r^2^) for correlation or regression tasks, and accuracy or AUROC for disease severity classification. When these were unavailable, we extracted free text outcomes. We extracted the best-performing metric for each AI model used for outcome reporting.

A quality assessment was performed by evaluating the presence of internal and external validation methods. Internal validation included cross-validation or split sample testing. External validation refers to using an independent dataset not used during the model training or testing phases to attempt to replicate the model’s performance. Risk of bias was assessed as per QUADAS-2 (Quality Assessment of Diagnostic Accuracy Studies 2) guidelines [6]. QUADAS-2 complements the data extraction process by evaluating four key domains: patient selection, index test, reference standard, and flow and timing of patients through the study. Each domain was assessed for risk of bias and review applicability concerns. The risk of bias was rated as low, high, or unclear based on specific criteria, while the review applicability was rated similarly based on how well the study aligned with the review question. Based on QUADAS-2 guidance, we tailored the index test interpretation to the unique challenges of AI diagnostic studies. Key concerns include data leakage, feature selection, adequacy of validation processes and data partitioning, and threshold identification.

## 3. Results

**Figure 1:**
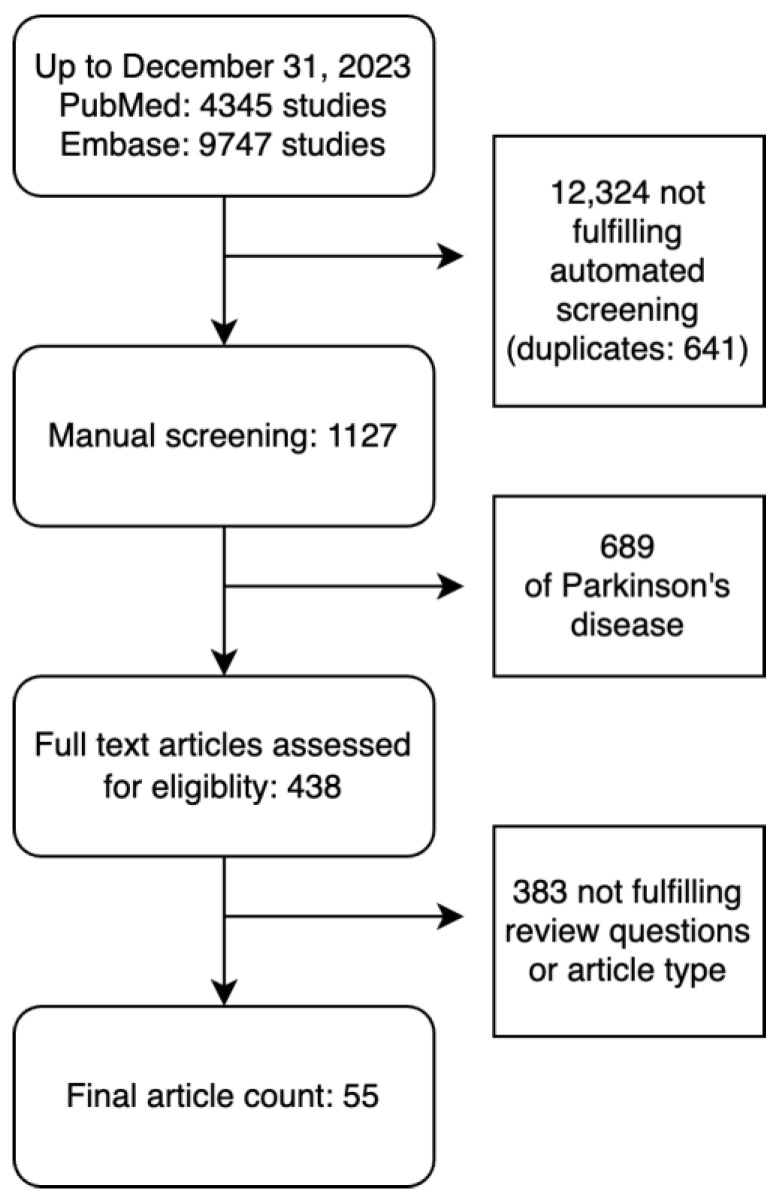
Flowchart of included studies.

The search strategy identified 14,092 studies published from 1990 to 2023. The automated title screener screened out a total of 12,324 studies without errors detected upon manual review. A manual screening of the remaining 1127 articles found 689 of PD, and an additional 383 abstracts, review articles, or studies not meeting review questions. Full-text assessment for eligibility resulted in 55 articles which met the review criteria (Figure 1) and underwent data extraction as well as quality and risk of bias appraisal. There were 13 articles on ataxia [7,8,9,10,11,12,13,14,15,16,17,18,19], 11 articles on chorea [20,21,22,23,24,25,26,27,28,29,30], five articles on dystonia [31,32,33,34,35], one article on tics [36], and 25 articles on tremor [37,38,39,40,41,42,43,44,45,46,47,48,49,50,51,52,53,54,55,56,57,58,59,60,61]. No articles on myoclonus were included. Thirty-five studies used machine learning (ML), sixteen used deep learning (DL), and four used both. The total number of subjects included was 11,946. For each hyperkinetic movement disorders group, ataxia had 2492 cases and 644 controls; chorea had 5256 cases and 271 controls; dystonia had 659 cases and 380 controls; tics had 11 cases with no controls; and tremor had 1540 cases and 693 controls. Data analyzed included 17 kinematics datasets (e.g., accelerometers, inertial measurement units), 15 studies included imaging-based datasets (e.g., MRI, fMRI, DAT scan), 12 studies included voice or video datasets (e.g., voice analysis, video analysis, eye tracking), and 10 studies included electrophysiology datasets (e.g., EEG, EMG), while the remainder included clinical examination and genetic datasets.

### 3.1. Disease Diagnosis and Quantitative Phenotyping Outcomes

Thirty-eight studies reported disease diagnosis outcomes (Table 1) and twenty-three reported quantitative phenotyping outcomes (Table 2). Six studies reported both types of outcomes and are featured in the tables below.

#### 3.1.1. Disease Diagnosis

Across all hyperkinetic movement disorders, accuracy was reported in 36 studies. The most used ML and DL algorithms were support vector machine (SVM, n = 13) and convolutional neural networks (CNN, n = 8), respectively. In ataxia (n = 8), data from an inertial measurement unit (IMU) was the most common dataset (n = 3), with 86–88% accuracy compared to clinical diagnosis for discriminating from healthy controls. In chorea (n = 8), data from MRI brain images were the most common dataset (n = 3), with accuracy ranging from 62% to 85% compared to clinical diagnosis for discriminating from healthy controls. In dystonia (n = 3), three studies used different datasets with accuracy for video analysis, MRI brain images, and voice recordings of 70%, 99%, and 65%, respectively, compared to clinical diagnosis for discriminating from healthy controls. For patients with tic disorder (n = 1), a video analysis dataset was used in one study, with an AUROC of 0.74 compared to clinical diagnosis. In tremor (n = 18), data from MRI brain images were the most often analyzed data (n = 4), with accuracy ranging from 58% to 98% and 100% compared to clinical diagnosis for discriminating from healthy controls (n = 3) and orthostatic tremor (n = 1), respectively.

#### 3.1.2. Quantitative Phenotyping

Across all hyperkinetic movement disorders, the correlation coefficient was reported in 10 studies. The most used ML and DL algorithms were SVM (n = 6) and CNN (n = 4), respectively. In ataxia (n = 7), the correlation coefficient ranged from 0.56 to 0.82 in four studies comparing IMU (n = 2), MRI brain images (n = 1), and video analysis datasets to clinical ratings; three remaining studies did not report a correlation coefficient. In chorea (n = 5), the correlation coefficient ranged from 0.66 to 0.77 in two studies comparing accelerometer and MRI/fMRI datasets to clinical ratings; three remaining studies did not report a correlation coefficient. In dystonia (n = 2), the correlation coefficient was 0.54–0.93 for two studies comparing video analysis to clinical ratings. In tremor (n = 9), the correlation coefficient ranged from 0.71 to 0.99 in two studies comparing IMU and MRI/fMRI datasets to clinical ratings; seven remaining studies did not report a correlation coefficient.

### 3.2. Quality Appraisal and Risk of Bias

All studies utilized internal validation methods (cross-validation, n = 47; split sample, n = 8). Only three studies documented external validation methods [8,29,49]. Five studies were judged as having “low risk of bias” [19,29,39,49,52], while the remaining studies had at least one category at risk of bias (Table 3). The index test was the most common category at risk of bias, meaning that the AI test results may have been interpreted with knowledge of the non-AI reference standard or that a threshold for disease diagnosis was not pre-specified. All studies demonstrated low concern regarding review applicability, indicating that their populations, index tests, and reference standards were highly relevant to the review PICO questions.

## 4. Discussion

In this study, we summarized the existing research literature applying AI methods to understand hyperkinetic movement disorders. We found 55 articles with 11,946 subjects that evaluated disease diagnosis and quantitative phenotyping. We implemented a semi-automated article screening pipeline that enhanced the reproducibility of results and utilized quality appraisal and risk of bias methods to evaluate selected studies. The most common ML and DL algorithms were SVM and CNN, respectively, and kinematics was the most frequent type of data analyzed with AI. The diagnostic accuracy was reported in 36 studies and ranged from 56 to 100% compared to clinical diagnoses, to differentiate patients with these conditions from healthy controls. The correlation coefficient was reported in 10 studies and ranged from 0.54 to 0.99 compared to clinical ratings for quantitative phenotyping. Importantly, all studies reported internal validation methods, but only three studies were validated with external data sets [8,29,49]. Only five studies were judged to have a low risk of bias [19,29,39,49,52], which is largely explained by the unclear methodology reporting of the index test interpretation. These quality concerns negatively impact the confidence in AI-based clinical research studies and the potential to integrate their findings into clinical practice.

We identified limitations with index test reporting that raise bias concerns. A major issue is data leakage, where training set information improperly influences the test set, often through normalization or feature engineering applied to the entire dataset rather than only to the training set. Inadequate validation processes, like insufficient cross-validation, resulted in overfitting, where models perform well on familiar data but poorly on unseen cases. Additionally, many studies provided insufficient detail on how training and testing sets were divided, which is needed for fair class representation. Furthermore, the determination of decision thresholds was often unclear. Selecting thresholds based on the training set can exacerbate overfitting, while thresholds derived from the testing set may misrepresent the model’s true diagnostic capabilities. Using QUADAS-2, similar limitations have been reported in AI-based imaging diagnostic studies across medical specialties [62]. In PD, concerns about AI-based research quality have also been raised [63], where in a study of 244 neuroimaging studies using AI methods for diagnosis, prognosis, or intervention, only 20% passed a set of minimal quality criteria. Data spillover, inadequate sample size, and insufficient biological plausibility were primary factors for quality loss [63]. Transparency in these practices is therefore needed to enhance the explainability of AI methods in diagnostic contexts [64], a critical requirement for its clinical implementation in movement disorders.

Another source of bias is the lack of external validation. AI diagnostic accuracy can be inflated by up to 30% due to overfitting the training and validation sets in a manner that would otherwise remain unnoticed without an external comparison [65]. Accuracy inflation can occur due to a bias towards the majority class (i.e., accuracy paradox), demographic imbalances associated with the disease of interest (i.e., second-order effects of the accuracy paradox), and the digital fingerprinting phenomenon (i.e., different samples from the same individual both in training and testing sets) [65]. Conversely, it is also possible that accuracy is deflated due to the imperfect nature of the comparison gold standard. Unlike in other specialties, such as oncology or cardiology, expert clinical examination by a trained movement disorders specialist remains the reference test (i.e., “gold standard”) in most hyperkinetic movement disorders. Only a few studies used genetic diagnosis as the reference test, which elevates the diagnostic certainty of the reference test. This is also applicable for studies quantifying disease severity, as severity ratings are anchored in clinical scales administered by clinicians.

There is a need to adopt AI-based research reporting guidelines that evaluate all the aspects of an AI pipeline: data collection, data preprocessing, feature selection, model training, and model testing. Several such guidelines are becoming increasingly available in recent years [66]. Although these guidelines emphasize medical imaging research and are not specific to movement disorders, some newer guidelines are being developed for diagnostic accuracy and prediction model studies. For example, STARD-AI and TRIPOD-AI are extensions of existing guidelines that may provide guidance relevant to movement disorders studies [66]. Ultimately, domain-specific guidelines will be needed to address AI-based kinematic and electrophysiology data analysis for accuracy and quantitative phenotyping in movement disorders. By adopting these measures, researchers can effectively reduce bias and enhance the fairness of AI models championing responsible AI [67].

This study has several limitations, including restricting the extraction of performance metrics to accuracy, correlation coefficient, determination coefficient, and AUROC. Other metrics, such as precision, recall, and F1 score for classification tasks, as well as mean absolute error and mean squared error for regression tasks, could provide additional information about the performance of AI algorithms. These metrics certainly speak to the clinical utility of research findings but were not considered, as evaluating method performance was not the focus of this review. We decided to prioritize the homogeneity of data extraction and focus on the most reported metrics available, which might best reflect the quality of methods, study design, and implementation. Another limitation in interpreting and comparing results across studies is the variability in data types (e.g., single vs. multi-channel, time-series vs. spatial-series), which dictate how a given AI method may be specifically implemented (e.g., a time-series CNN to evaluate IMU data vs. a 2D or 3D CNN to evaluate MRI data). These methodological differences can significantly affect model performance, making direct comparisons across studies challenging. Hence, we chose to aggregate data by dataset and not AI method. Another limitation of this review is the use of an automated article screening pipeline. This, in principle, increases reproducibility, as it reduces human error in title screening, which was needed due to the large number of articles found with broad search terms, but it may have overlooked relevant studies with missing keywords in the title. We aimed to reduce this caveat by randomly screening a sample of these articles, finding no inadequately excluded articles by the automated title screener. However, only 20% of titles were evaluated in our random screening, so it is possible that articles that were not manually reviewed were incorrectly excluded. Finally, QUADAS-2 is not tailored to evaluate bias in accuracy studies powered by AI, possibly conflating the perceived risk of bias. To our knowledge, no specific guidelines exist for this task; QUADAS-AI is under development [68]. These guidelines should highlight reporting critical steps of AI model development and testing, including data collection and preprocessing, model selection and computational considerations, model training procedure, model evaluation, and model generalizability and interpretability.

## 5. Conclusions

This systematic review demonstrates a growing enthusiasm for research using AI algorithms to investigate hyperkinetic movement disorders, most commonly for diagnosis and prognosis, tracking disease progression, and predicting outcomes. Datasets were analyzed across ataxia, chorea, dystonia, tics, and tremors, and methods were implemented, comparing them against ground-truth assessments to characterize diagnostic and quantitative phenotyping performance. In 36 studies, diagnostic accuracy ranged from 56% to 100%, while ten studies reported correlation coefficients from 0.54 to 0.99 for quantitative phenotyping. Performance metric reporting was heterogeneous: accuracy was often reported, while correlation coefficient was seldom reported. Most studies had at least one feature at high risk of bias, possibly due to inadequate reporting or intrinsic research design flaws. Our results highlight the need to adopt rigorous AI-based research guidelines to minimize reporting heterogeneity and bolster the clinical interpretability of results from these studies and, ultimately, their clinical translation.

## Figures and Tables

**Table 1:** Studies using AI for disease diagnosis.

First Author	Dataset	Cases	Controls	Index Test	Reference Test	Outcome
**ATAXIA**						
Dominguez-Vega Z.T [7]	IMU	22 Ataxia, 14 Dyspraxia	24 HC	RF	Clinical diagnosis	86% accuracy
Ngo T [8]	IMU	62 Ataxia	24 HC	GNB	Clinical diagnosis	88% accuracy
Ngo T [9]	IMU	103 Ataxia	69 HC	CNN *	Clinical diagnosis	86% accuracy
Schultz B.G [10]	Voice recordings	112 MS, 73 FA	229 HC	CatBoost	Clinical diagnosis	82% accuracy
Kashyap B [11]	Voice recordings	22 CANVAS, 21 FA, 20 SCA3	28 HC	SVM	Clinical diagnosis	95% accuracy
Nunes A.S [12]	Video analysis	169 Ataxia	78 PD, 58 HC	DeepNMA *	Clinical diagnosis	AUROC 0.92
Rojas F [13]	Electrooculography	10 SCA2	10 HC	KM	Clinical diagnosis	100% accuracy
Hernandez-Castillo C.R [14]	fMRI images	26 SCA7	26 HC	SVM	Genetic testing	92% accuracy
**CHOREA**						
Klöppel S [20]	MRI brain images	96 Pre-HD	95 HC	SVM	Clinical diagnosis	69% accuracy
Lavrador R [21]	MRI brain images	14 Pre-HD, 11 HD	18 HC	SVM	Clinical diagnosis	85% accuracy
Rizk-Jackson A [22]	fMRI and MRI images	39 Pre-HD	25 HC	SVM, LDA	Clinical diagnosis	SVM: 62% accuracy LDA: 76% accuracy
de Tommaso M [23]	EEG	13 HD, 7 Pre-HD	13 HC	ANN *	Clinical diagnosis and rating	88% accuracy
Odish OFF [24]	EEG	20 HD, 6 Pre-HD	25 HC	SPR	Clinical rating	83% accuracy
Bennasar M [25]	Accelerometer	44 HD	48 HC	EC	Genetic diagnosis	99% accuracy
Mannini A [26]	IMU	17 HD	15 Stroke, 10 HC	SVM, HMM	Clinical diagnosis	SVM: 91% accuracy HMM: 86% accuracy
Miranda Â [27]	Eye-tracking video camera	14 Pre-HD, 14 HD	22 HC	SVM	Clinical diagnosis	73% accuracy
**DYSTONIA**						
Loram I [31]	Video analysis	35 CD	26 HC	U-Net *	Clinical diagnosis	70% accuracy
Valeriani D [32]	MRI brain images	392 DYS	220 HC	CNN *	Clinical diagnosis	99% accuracy
Yao Y [33]	Voice recordings	44 AdLD, 45 AbLD, 45 voice tremor	130 HC	CNN *	Clinical diagnosis	65% accuracy
**TICS**						
Conelea C [36]	Video analysis	11 TS	0	CNN *	Clinical diagnosis	0.74 AUROC
**TREMOR**						
Benito-León J [37]	MRI brain images	15 ET	14 OT	SVM	Clinical diagnosis	100% accuracy
Zhang X [38]	fMRI and MRI images	162 ET	153 HC	SVM	Clinical diagnosis	61% accuracy
Zheng X [39]	fMRI and MRI images	20 ET	5 HC	CNN *	Clinical diagnosis	89–98% accuracy
Serrano JI [40]	MRI brain images	18 ET	18 HC	SVM, NB, RI, KNN, ANN *	Clinical diagnosis	SVM: 58% accuracy NB: 80% accuracy RI: 61% accuracy KNN: 72% accuracy ANN: 75% accuracy
Li Q [41]	fMRI images	101 ET	105 HC	SVM, LR, RF, NB	Clinical diagnosis	SVM: 85% accuracy LR: 85% accuracy RF: 85% accuracy NB: 76% accuracy
Ferreira G.A.S. [42]	IMU	10 ET	34 PD	KNN, DT, RF, NB, SVM	Clinical rating and wrist gyroscope	KNN: 93% accuracy DT: 86% accuracy RF: 87% accuracy NB: 93% accuracy SVM: 86% accuracy
Ma C [43]	IMU	54 ET	0	SVM, KNN, NB, TE	Clinical rating	SVM: 98% KNN: 97% NB: 94% TE: 99%
Pascual-Valdunciel A [44]	IMU	12 ET	11 HC	KNN, SVM, RF, LSTM *	Clinical diagnosis	KNN: 94% accuracy SVM: 91% accuracy RF: 95% accuracy LSTM: 96% accuracy
Piepjohn P [45]	Accelerometer and sEMG	561 Tremor	0	DT, ANN *	Clinical diagnosis	DT: 83% accuracy ANN: 84% accuracy
Hossen A [46]	Accelerometer and sEMG	41 ET	39 PD	ANN *	Clinical diagnosis	92% accuracy
Palmes P [47]	sEMG	20 Tremor	18 HC	SVM	Clinical diagnosis and sEMG	98% accuracy
Samaee S [48]	sEMG	11 Tremor	0	HMM	sEMG	81% accuracy
Balachandar A [49]	Accelerometer	19 PD, 42 ET, 17 DysT	6 HC	RF, LR	Clinical diagnosis	RF: 78% LR: NR
Suppa A [50]	Voice recordings	58 ET	74 HC	SVM	Clinical diagnosis	80–98% accuracy
Wang X [51]	Video analysis	50 Tremor	5 HC	SVM, LSTM *, CNN-LSTM *	Clinical rating	SVM: 56% accuracy LSTM: 80% accuracy CNN-LSTM: 81% accuracy
Wang Y [52]	Handwriting samples	50 ET	40 HC	CNN *	Clinical diagnosis	89% accuracy
Lopez-de-Ipina K [53]	Handwriting samples	29 ET	21 HC	SVM, KNN, MLP	Clinical diagnosis	SVM: 82% accuracy KNN: 79% accuracy MLP: 88% accuracy
Hamilton D [54]	DAT scan	18 ET and PD	NR	ANN *	Radiological diagnosis	100% accuracy

AdLD, Adductor Laryngeal Dystonia; AbLD, Abductor Laryngeal Dystonia; ANN, Artificial Neural Network; BLVA, Bayesian Latent Variable Analysis; CD, Cervical Dystonia; CNN, Convolutional Neural Network; DAT, dopamine active transporter; DT, Decision Tree; DysT, Dystonic Tremor; DYS, Dystonia; ECoG, Electrocorticography; EC, Ensemble Classifier; ET, Essential Tremor; FA, Friedreich Ataxia; HD, Huntington’s Disease; HC, Healthy Control; HMM, Hidden Markov Model; IMU, Inertial Measurement Unit; KM, K-Means; KNN, K-Nearest Neighbor; LaR, Lasso Regression; LDA, Linear Discriminant Analysis; LiR, Linear Regression; LR, Logistic Regression; LSTM, Long Short-Term Memory; MLP, Multilayer Perceptron; MS, Multiple Sclerosis; MV, Majority Voting; NB, Naïve Bayes; NR, Not Reported; OT, Orthostatic Tremor; PD, Parkinson’s Disease; Pre-HD, Pre-symptomatic Huntington’s Disease; RF, Random Forest; RI, Rule Induction; RVM, Relevance Vector Machine; SCA2, Spinocerebellar Ataxia Type 2; SCA3, Spinocerebellar Ataxia Type 3; SCA7, Spinocerebellar Ataxia Type 7; sEMG, surface electromyography; SPR, Statistical Pattern Recognition; SVM, Support Vector Machines; TE, Tree Ensemble; TS, Tourette Syndrome; U-Net, U-Net Architecture; XGBoost, Extreme Gradient Boosting. * Denotes deep learning algorithm.

**Table 2:** Studies using AI for Quantitative Phenotyping.

First Author	Dataset	Cases	Controls	Index Test	Reference Test	Outcome
**ATAXIA**						
Krishna R [16]	IMU	39 Ataxia	31 HC	LDA	Clinical diagnosis	r = 0.77–0.82 with rating score
Ngo T [8]	IMU	62 Ataxia	24 HC	GNB	Clinical rating	r = 0.72 with rating subscore
Kadirvelu B [19]	IMU	9 FA	9 HC	SVM, DT, LR, KNN, RF	Clinical rating	Severity prediction 9 months in the future was 1.7–4 times more precise than with clinical rating
Hu J [15]	MRI brain images	66 SCA3	58 HC	RVM	Clinical rating	r = 0.56 with rating score
Nunes A.S [12]	Video analysis	169 Ataxia	78 PD, 58 HC	DeepNMA *	Clinical rating	r = 0.64–0.67 with rating score
Ru D [17]	Genetics	1008 SCA3	0	ANN *	Clinical rating	R^2^ of 0.653 predicting age at onset
Ru D [18]	Natural history SCA3 Dataset	716 SCA3	0	SVM	Clinical rating	76% accuracy to predict within 10% of the clinical rating
**CHOREA**						
Ko J [28]	HD natural history datasets	4961 HD	0	KM	Clinical rating	Age and polyglutamine repeat length at enrollment top features for severity clustering
Mohan A [29]	HD natural history datasets	1942 HD, 1215 Pre-HD	0	BLVA, HMM	Clinical rating	Nine clusters segregation for increasing severity
Bennasar M [25]	Accelerometer	44 HD	48 HC	LiR	Clinical rating	r = 0.77 with rating score
Rizk-Jackson A [22]	fMRI and MRI images	39 Pre-HD	25 HC	SVR	Clinical rating	Caudate and caudate r = 0.66 for MRI; insular cortex r = 0.68 for fmri
Zhang S [30]	Gait kinematics	12 HD	0	CNN *	Clinical rating	89% accuracy for high vs. low severity
**DYSTONIA**						
Vu J.P [34]	Video analysis	93 CD	n/a	LiR	Clinical rating	r = 0.54 with rating score
Yousef AM [35]	Video analysis	5 AdLD	4 HC	CNN *, U-Net	Clinical rating	r = 0.93 with rating score
**TREMOR**						
Bianco MG [55]	MRI brain images	66 ET	45 HC	XGBoost	Clinical rating	ET with rest tremor had increased roughness and mean curvature in frontotemporal areas compared with HC and ET
Prasad S [59]	MRI brain images	40 ET	37 HC	XGBoost, RF, SVM	Clinical diagnosis	ET vs. HC had atrophy of middle and inferior cerebellar peduncle
Purrer V [61]	MRI brain images	61 ET	29 PD	CNN *	Clinical diagnosis	ET without rest tremor had thalamic atrophy vs. tremor-predominant PD
Saccà V [60]	fMRI images	18 ET	19 HC	SVM	Clinical rating	AUROC 0.75 for four networks (language, primary visual, cerebellum, and attention)
Zheng X [39]	fMRI and MRI images	20 ET	5 HC	CNN *	Clinical rating	r = 0.92 to rating scores
Gao Y [56]	Genetics	32 ET	20 HC	LaR, SVM	Clinical rating	AUROC >0.7 for APOE, SENP6, and ZNF148.
Houston B [57]	ECoG strip data	3 ET	0	LR	Wrist gyroscope	75% accuracy to classify five prompted tasks
Pascual-Valdunciel A [58]	IMU	12 ET	0	LSTM *	Clinical rating	r = 0.71- 0.99 correlation with rating score
Wang Y [52]	Handwriting samples	50 ET	40 HC	CNN *	Clinical rating	76–83% accuracy of subregional handwriting differences

AdLD, Adductor Laryngeal Dystonia; AbLD, Abductor Laryngeal Dystonia; ANN, Artificial Neural Network; AUROC, Area Under the Receiver Operating Characteristic curve; BLVA, Bayesian Linear Variational Approximation; CD, Cervical Dystonia; CNN, Convolutional Neural Network; DT Decision Tree; DYS, Dystonia; ECoG, Electrocorticography; EC, Ensemble Classifier; ET, Essential Tremor; FA, Friedreich Ataxia; fMRI, Functional Magnetic Resonance Imaging; HC, Healthy Control; HD, Huntington’s Disease; IMU, Inertial Measurement Unit; KM, K-Means; LaR, Lasso Regression; LDA, Linear Discriminant Analysis; LiR, Linear Regression; LR, Logistic Regression; LSTM, Long Short-Term Memory; MRI, Magnetic Resonance Imaging; MS, Multiple Sclerosis; MV, Majority Voting; NB, Naïve Bayes; NR, Not Reported; OT, Orthostatic Tremor; PD, Parkinson’s Disease; Pre-HD, Pre-symptomatic Huntington’s Disease; RF, Random Forest; RVM, Relevance Vector Machine; SCA2, Spinocerebellar Ataxia Type 2; SCA3, Spinocerebellar Ataxia Type 3; SCA7, Spinocerebellar Ataxia Type 7; sEMG, surface electromyography; SPR, Statistical Pattern Recognition; SVM, Support Vector Machine; TE, Tree Ensemble; TS, Tourette Syndrome; U-Net, U-Net Architecture; XGBoost, Extreme Gradient Boosting; r, correlation coefficient; r², R-squared determination coefficient.* denotes deep learning algorithm.

**Table 3:** QUADAS-2 Summary Findings.

First Authors	Risk of Bias				Applicability Concerns		
	Patient Selection	Index Test	Reference Standard	Flow and Timing	Patient Selection	Index Test	Reference Standard
**ATAXIA**							
Dominguez-Vega Z.T [7]	Low	High	Low	Low	Low	Low	Low
Hernandez-Castillo C.R [14]	Low	High	Low	High	Low	Low	Low
Hu J [15]	Low	High	Low	Low	Low	Low	Low
Kashyap B [11]	Low	High	Low	Low	Low	Low	Low
Krishna R [16]	Low	High	Low	Low	Low	Low	Low
Ngo T [8]	Low	High	Low	Low	Low	Low	Low
Ngo T [9]	Low	High	Low	Low	Low	Low	Low
Nunes A.S [12]	Low	High	Low	Low	Low	Low	Low
Rojas F [13]	Low	High	Low	Low	Low	Low	Low
Ru D [17]	Low	High	Low	Low	Low	Low	Low
Ru D [18]	Low	High	Low	Low	Low	Low	Low
Kadirvelu B [19]	Low	Low	Low	Low	Low	Low	Low
Schultz B.G [10]	Low	High	Low	Low	Low	Low	Low
**CHOREA**							
Bennasar M [25]	Low	High	Low	Low	Low	Low	Low
de Tommaso M [23]	Low	High	Low	Low	Low	Low	Low
Klöppel S [20]	Low	High	Low	Low	Low	Low	Low
Ko J [28]	Low	High	Low	Low	Low	Low	Low
Lavrador R [21]	Low	High	Low	Low	Low	Low	Low
Mannini A [26]	Low	High	Low	Low	Low	Low	Low
Miranda Â [27]	Low	High	Low	Low	Low	Low	Low
Mohan A [29]	Low	Low	Low	Low	Low	Low	Low
Odish OFF [24]	Low	High	Low	Low	Low	Low	Low
Rizk-Jackson A [22]	Low	High	Low	High	Low	Low	Low
Zhang S [30]	Low	High	Low	High	Low	Low	Low
**DYSTONIA**							
Loram I [31]	Low	High	Low	Low	Low	Low	Low
Valeriani D [32]	Low	High	Low	Low	Low	Low	Low
Vu J.P [34]	Low	High	Low	High	Low	Low	Low
Yao Y [33]	Low	High	Low	Low	Low	Low	Low
Yousef AM [35]	Low	High	Low	Low	Low	Low	Low
**TICS**							
Conelea C [36]	Low	High	Low	Low	Low	Low	Low
**TREMOR**							
Benito-León J [37]	Low	High	High	Low	Low	Low	Low
Bianco MG [55]	Low	High	Low	Low	Low	Low	Low
Gao Y [56]	Low	High	Low	Low	Low	Low	Low
Houston B [57]	Low	High	Low	Low	Low	Low	Low
Li Q [41]	Low	High	Low	Low	Low	Low	Low
Lopez-de-Ipina K [53]	Low	High	Low	Low	Low	Low	Low
Ma C [43]	Low	High	Low	Low	Low	Low	Low
Palmes P [47]	Low	High	Low	Low	Low	Low	Low
Pascual-Valdunciel A [44]	Low	High	Low	Low	Low	Low	Low
Pascual-Valdunciel A [58]	Low	High	Low	Low	Low	Low	Low
Piepjohn P [45]	Low	High	Low	Low	Low	Low	Low
Prasad S [59]	Low	High	Low	Low	Low	Low	Low
Saccà V [60]	Low	High	Low	Low	Low	Low	Low
Samaee S [48]	Low	High	Low	Low	Low	Low	Low
Suppa A [50]	Low	High	Low	Low	Low	Low	Low
Wang X [51]	Low	High	Low	Low	Low	Low	Low
Wang Y [52]	Low	Low	Low	Low	Low	Low	Low
Zhang X [38]	Low	High	Low	Low	Low	Low	Low
Zheng X [39]	Low	Low	Low	Low	Low	Low	Low
Balachandar A [49]	Low	Low	Low	Low	Low	Low	Low
Serrano JI [40]	Low	High	Low	Low	Low	Low	Low
Ferreira G.A.S. [42]	Low	High	Low	Low	Low	Low	Low
Hamilton D [54]	Low	High	Low	Low	Low	Low	Low
Hossen A [46]	Low	High	Low	Low	Low	Low	Low
Purrer V [61]	Low	High	Low	Low	Low	Low	Low

Patient selection evaluates the representativeness of the study population and any inappropriate exclusions; Index test examines the execution and interpretation of the diagnostic test for potential biases; Reference standard reviews the reliability of the standard used to classify the condition; Flow and timing analyzes patient flow through the study and the timing of tests. A “low” concern rating indicates that the study is likely free from bias and aligns well with the review question, while a “high” concern rating suggests significant potential for bias or issues that could affect the validity of the study’s findings.

## Supplementary Materials

The following supporting information can be downloaded at: https://www.mdpi.com/article/10.3390/jcm13237009/s1, Table S1. Search Terms for Embase And Pubmed.
