# Supplementary material for: Artificial Intelligence in the Diagnosis and Quantitative Phenotyping of Hyperkinetic Movement Disorders: A Systematic Review
Source: J Clin Med. 2024 Nov 21;13(23):7009. doi: 10.3390/jcm13237009 (PMC11642074; doi:10.3390/jcm13237009)
Supplement: Supplementary file 1 [file jcm-13-07009-s001.zip › jcm-3324582-supplementary.pdf]

Journal of
Clinical Medicine

MDPI

Table S1. Search Terms for Embase And Pubmed.

Embase

Session Results

....

No.	Query Results	Results	Date
#2.	#1 AND [embase]/lim	9,747	31 Dec 2023
#1.	('artificial intelligence'/exp OR 'machine learning'/exp OR 'unsupervised machine learning'/exp OR 'deep learning'/exp OR 'artificial intelligence' OR 'machine learning' OR 'deep learning') AND ('motor dysfunction'/exp OR 'parkinson disease'/exp OR 'dystonia'/exp OR 'dystonic disorder'/exp OR 'huntington chorea'/exp OR 'essential tremor'/exp OR 'ataxia'/exp OR 'diffuse lewy body disease'/exp OR 'shy drager syndrome'/exp OR 'progressive supranuclear palsy'/exp OR 'movement disorders' OR 'parkinsons' OR 'dystonia' OR 'huntingtons' OR 'essential tremor' OR 'ataxia' OR ‘myoclonus’ OR 'progressive supranuclear palsy' OR 'psp' OR 'msa' OR 'tic'/exp OR 'gilles de la tourette syndrome'/exp OR 'tics' OR 'tourette')	11,110	31 Dec 2023

....

PubMed

4,345 results 31 Dec 2023

'("Artificial Intelligence"[Mesh] OR "Machine Learning"[Mesh] OR "Unsupervised Machine Learning"[Mesh] OR "Deep Learning"[Mesh] OR artificial intelligence OR machine learning OR deep learning) AND ("Movement Disorders"[Mesh] OR "Parkinson Disease"[Mesh] OR "Dystonia"[Mesh] OR "Dystonic Disorders"[Mesh] OR "Huntington Disease"[Mesh] OR "Essential Tremor"[Mesh] OR "Ataxia"[Mesh] OR "Lewy Body Disease"[Mesh] OR "Multiple System Atrophy"[Mesh] OR "Supranuclear Palsy, Progressive"[Mesh] OR movement disorders OR parkinsons OR dystonia OR huntingtons OR essential tremor OR ataxia OR myoclonus OR progressive supranuclear palsy OR psp OR msa OR "Tics"[Mesh] OR "Tourette Syndrome"[Mesh] OR tics OR tourette)'
